# Assessing Confidence in Root Placement on Phylogenies: An Empirical Study Using Nonreversible Models for Mammals

**DOI:** 10.1093/sysbio/syab067

**Published:** 2021-08-13

**Authors:** Suha Naser-Khdour, Bui Quang Minh, Robert Lanfear

**Affiliations:** 1 Department of Ecology and Evolution, Research School of Biology, Australian National University, Canberra, Australian Capital Territory, Australia; 2 Research School of Computer Science, Australian National University, Canberra, Australian Capital Territory, Australia

## Abstract

Using time-reversible Markov models is a very common practice in phylogenetic analysis, because although we expect many of their assumptions to be violated by empirical data, they provide high computational efficiency. However, these models lack the ability to infer the root placement of the estimated phylogeny. In order to compensate for the inability of these models to root the tree, many researchers use external information such as using outgroup taxa or additional assumptions such as molecular clocks. In this study, we investigate the utility of nonreversible models to root empirical phylogenies and introduce a new bootstrap measure, the *rootstrap*, which provides information on the statistical support for any given root position. [Bootstrap; nonreversible models; phylogenetic inference; root estimation.]

The most widely used method for rooting trees in phylogenetics is the outgroup method. Although the use of an outgroup to root an unrooted phylogeny usually outperforms other rooting methods (Huelsenbeck et al. 2002), the main challenge with this method is to find an appropriate outgroup (Watrous and Wheeler 1981; Maddison et al. 1984; Smith 1994; Swofford et al. 1996; Lyons-Weiler et al. 1998; Milinkovitch and Lyons-Weiler 1998). Outgroups that are too distantly related to the ingroup may have substantially different molecular evolution than the ingroup, which can compromise accuracy. And outgroups that are too closely related to the ingroup may not be valid outgroups at all.

It is possible to infer the root of a tree without an outgroup using molecular clocks (Huelsenbeck et al. 2002; Drummond et al. 2006). A strict molecular clock assumes that the substitution rate is constant along all lineages, a problematic assumption especially when the ingroup taxa are distantly related such that their rates of molecular evolution may vary. Relaxed molecular clocks are more robust to deviations from the clock-like behavior (Drummond et al. 2006), although previous studies have shown that they can perform poorly in estimating the root of a phylogeny when those deviations are considerable (Tria et al. 2017).

Other rooting methods rely on the distribution of branch lengths, including Midpoint Rooting (Farris 1972), Minimal Ancestor Deviation (Tria et al. 2017), and Minimum Variance Rooting (Mai et al. 2017). Such methods also assume a clock-like behavior; however, they are less dependent on this assumption as the unrooted tree is estimated without it. Similar to inferring a root directly from molecular clock methods, the accuracy of those rooting methods decreases with higher deviations from the molecular clock assumption (Mai et al. 2017).

Other less common rooting methods that can be used in the absence of outgroup are: rooting by gene duplication (Dayhoff and Schwartz 1980; Gogarten et al. 1989; Iwabe et al. 1989), indel-based rooting (Rivera and Lake 1992; Baldauf and Palmer 1993; Lake et al. 2007), rooting the species tree from the distribution of unrooted gene trees (Allman et al. 2011; Yu et al. 2011), and probabilistic coestimation of gene trees and species tree (Boussau et al. 2013).

All the methods mentioned above, apart from the molecular clock, infer the root position independently of the ML tree inference. The only existing approach to include root placement in the ML inference is the application of nonreversible models. Using nonreversible substitution models relaxes the fundamental assumption of time-reversibility that exists in the most widely used models in phylogenetic inference (Jukes and Cantor 1969; Kimura 1980; Hasegawa et al. 1985; Tavaré 1986; Dayhoff et al. 1978; Jones et al. 1992; Tamura and Nei 1993; Whelan and Goldman 2001; Le and Gascuel 2008). This in itself is a potentially useful improvement in the fit between models of sequence evolution and empirical data. In addition, since nonreversible models naturally incorporate a notion of time, the position of the root on the tree is a parameter that is estimated as part of the ML tree inference. Since the incorporation of nonreversible models in efficient ML tree inference software is relatively new (Minh et al. 2020), we still understand relatively little about the ability of nonreversible models to infer the root of a phylogenetic tree, although a recent simulation study has shown some encouraging results (Bettisworth and Stamatakis 2020).

Regardless of the rooting method and the underlying assumptions, it is crucial that we are able to estimate the statistical confidence we have in any particular placement of the root on a phylogeny. A number of previous studies have sensibly used ratio likelihood tests such as the Shimodaira–Hasegawa (SH) test (Shimodaira and Hasegawa 1999) and the approximately unbiased (AU) test (Shimodaira 2002) to compare a small set of potential root placements, rejecting some alternative root placements in favor of the ML root placement (e.g., Nardi et al. 2003; Steenkamp et al. 2006; Jansen et al. 2007; Moore et al. 2007; Williams et al. 2010; Kocot et al. 2011; Zhou et al. 2011; Whelan et al. 2015; Zhang et al. 2018), these tests are still somewhat limited in that they do not provide the level of support the data have for a certain root position.

There is strong empirical evidence that molecular evolutionary processes are rarely reversible (Squartini and Arndt 2008; Naser-Khdour et al. 2019), but few studies have explored the accuracy of nonreversible substitution models to root phylogenetic trees (Huelsenbeck et al. 2002; Yap and Speed 2005; Williams et al. 2015; Cherlin et al. 2018; Bettisworth and Stamatakis 2020). Most studies that have looked at this question in the past have focused on either simulated data sets (Huelsenbeck et al. 2002; Jayaswal et al. 2011; Cherlin et al. 2018) or relatively small empirical data sets (Yang and Roberts 1995; Yap and Speed 2005; Jayaswal et al. 2011; Heaps et al. 2014; Williams et al. 2015; Cherlin et al. 2018). In both cases, the addressed substitution models were nucleotide models, and to our knowledge, no study has yet investigated the potential of amino acid substitution models in inferring the root placement of phylogenetic trees.

In this article, we focus on evaluating the utility of nonreversible amino acid and nucleotide substitution models to root the trees, and we introduce a new metric, the *rootstrap support value*, which estimates the extent to which the data support every possible branch as the placement of a root in a phylogenetic tree. Unlike previous studies that used Bayesian methods with nonreversible substitution models to infer rooted ML trees (Heaps et al. 2014; Cherlin et al. 2018), we will conduct our study in a maximum likelihood (ML) framework using IQ-TREE (Minh et al. 2020). A clear advantage of ML over the Bayesian analysis is that there is no need for a prior on the parameter distributions, which sometimes can affect tree inference (Huelsenbeck et al. 2002; Cherlin et al. 2018). Even though estimating the nonreversible model’s parameters by maximizing the likelihood function seems more computationally intensive than calculating posterior probabilities (Huelsenbeck et al. 2002), the IQ-TREE algorithm is sufficiently fast to allow us to estimate root placements, with *rootstrap support* for very large data sets.

A recent study investigated the ability of nonreversible nucleotide models to infer the root placement of phylogenetic trees (Bettisworth and Stamatakis 2020). This study showed that IQ-TREE performs competitively with a new rooting tool, RootDigger. In most simulated data sets, IQ-TREE slightly outperformed RootDigger in terms of root placements, but no comparisons were made between RootDigger and IQ-TREE on empirical data sets. Although RootDigger is significantly faster than IQ-TREE (Bettisworth and Stamatakis 2020), the former is limited to nucleotide substitution models. Since we are interested in both nucleotide and amino acid nonreversible models, we used IQ-TREE for tree and root inference in this study.

## Materials and Methods

### The “Rootstrap” Support, and Measurements of Error in Root Placement

**Figure 1.**
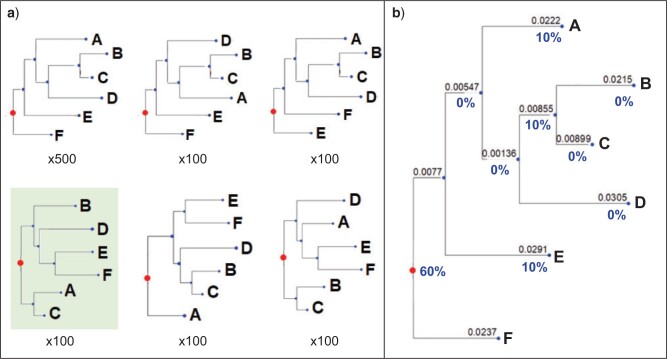
Illustration of the rootstrap concept. a) The bootstrap replicates trees. b) The ML tree with the rootstrap support values for each branch. Note that the sum of the rootstrap support values is less than 100% due to 100 bootstrap replicates trees (green box, in the lower-left corner) that have their root at a branch that does not exist in the ML tree.

To compute rootstrap supports, we conduct a bootstrap analysis, that is, resampling alignment sites with replacement, to obtain a number of bootstrap trees. We define the *rootstrap* support for each branch in the ML tree, as the proportion of bootstrap trees that have the root on that branch. Since the root can be on any branch in a rooted tree, the rootstrap support values are computed for all the branches including external branches. The sum of the rootstrap support values along the tree are always smaller than or equal to one. A sum that is smaller than one can occur when one or more bootstrap replicates are rooted on a branch that does not occur in the ML tree (Fig. 1).

By definition, the rootstrap support values for internal branches are bounded by the bootstrap support values at those branches. On the other hand, the rootstrap support values for tips (leaf branches) are bounded by 100%, as tips always appear in all the bootstrap trees.

**Figure 2.**
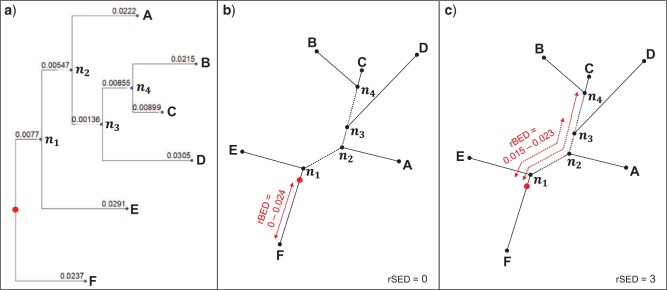
An example to illustrate the root error distance. a) the ML rooted tree, b) the root branch-length error distance (rBED) if the true root is believed to be on the same ML root branch (rSED $=$ 0), c) the rBED if the true root is believed to be on the branch between D and the clade of C $+$ B (rSED $=$ 3).

If the true position of the root is known (e.g., in simulation studies) or assumed (e.g., in the empirical cases we present below), we can calculate additional measurements of the error of the root placement. We introduce two such measurements here: *root branchlength error distance* (rBED) and *root split error distance* (rSED). Since the nonreversible model infers the exact position of the root on a branch, we define the *root branchlength error distance* (rBED) as the range between the minimum and maximum distance between the inferred root position and the “true root” branch. If the true root is on the same branch as the ML tree root, then rBED will be between 0 and the distance between the ML tree root and the farthest point on that branch (Fig. 2). Since rBED is based on branch lengths only, it ignores the absolute number of splits between the ML tree root and the true root; and therefore, the rBED for the true root being on the same ML root branch can be bigger than the rBED for the true root being on a different branch (e.g., Fig. 2). In order to account for the number of splits (nodes) between the ML tree root and the true root, we define *root split error distance* (rSED) as the number of splits between the ML root branch and the branch that is believed to contain the true root (Fig. 2).

The rootstrap, rBED, and rSED assess different aspects of the root placement. While the rootstrap offers an indication of the support that the data have for a certain branch to be the root branch, rBED and rSED provide an estimation to the accuracy of the method in estimating the exact root position if the root position is known or assumed in advance. In other words, the rootstrap value is a measure for the robustness of the root placement given the model and the data and can be used on any data set regardless of whether the true root position is known, while rBED and rSED are measures of the accuracy of the nonreversible model to find the root placement given the data, and require the root position to be known or assumed in advance.

### Empirical Data Sets

Because nonreversible amino acid models require the estimation of a large number of parameters, and because we suspected that the information in any such analysis on the placement of the root branch of a tree might be rather limited, we searched for empirical data sets that met a number of stringent criteria:

1. Existence of both DNA and amino acid multiple sequences alignments (MSA) for the same loci.
2. Genome-scale MSAs to ensure that the MSAs have as much information as possible with which to estimate the nonreversible models’ free parameters and the root position. Since we do not know the number of sites required to correctly infer the rooted ML tree, we define 100,000 sites as the minimum number of required sites. This also allows us to subsample the data set to explore the ability of smaller data sets to infer root positions.
3. Highly curated alignments: since the quality of the inferred phylogeny is highly dependent on the quality of the MSA (Philippe et al. 2011), we focused on data sets that were highly curated for misalignment, contamination, and paralogy.
4. Existence of several clades for which there is a very strong consensus regarding their root placement. Since we are interested in evaluating the performance of nonreversible models to infer root placements in an empirical rather than a simulation context, we need to identify monophyletic subclades for which we can be almost certain about their root position. This enables us to divide the data set into nonoverlapping subclades for which we are willing to assume we know the root positions. Furthermore, we define the minimum number of taxa in each subdata set as five.
  We initially identified a number of genome-scale data sets that contained large numbers of nucleotide and amino acid MSAs. In many cases, it was difficult to determine whether these alignments had been rigorously curated, and even more challenging to find data sets for which the root position of a number of subclades could be assumed with confidence. The only data set that met all of our criteria was a data set of placental mammals with 78 ingroup taxa and 3,050,199 amino acids (Wu et al. 2019). This data set was originally published as an MSA (Liu et al. 2017) based on very high-quality sequences from Ensembl, NCBI, and GenBank databases. After receiving detailed critiques for potential alignment errors (Gatesy and Springer 2017), the data set was further processed to remove potential sources of bias and error, and an updated version of the data set was recently published (Wu et al. 2018). The fact that this alignment comes from one of the most well-studied clades on the planet, has been independently curated and critiqued by multiple groups of researchers and includes truly genome-scale data, makes it ideally suited for our study. The curated alignments can be found on figshare (https://figshare.com/s/622e9e0a156e5233944b) under the name “Wu_2018_aa” and “Wu_2018_dna” for the amino-acid and nucleotide alignments, respectively.

### Selecting Clades with a Well-Defined Root

Since our main objective in this study is to evaluate the effectiveness of nonreversible models and the rootstrap value in estimating and measuring the support for a given root placement on empirical data sets, we must identify a collection of subclades of the larger mammal data set for which it is reasonable to assume a root position. We acknowledge, of course, that outside a simulation framework it is not possible to be certain of the root position of a clade. Nevertheless, it is possible to identify clades for which the position of the root is well supported and noncontroversial, thus minimizing the chances that the assumption of a particular root position is incorrect. To achieve this, we analyzed the root position of each order and superorder in the data set, and defined “*well-defined clades*” that fulfilled *all* of the following criteria:

(1) It contains at least five taxa. This ensures that the probability of obtaining a random ML rooted tree to be at most 0.95%. For clades with four taxa, there are 15 different rooted topologies, and therefore a 6.7% probability to get any particular root position by chance. On the other hand, for clades with at least five taxa, there are at least 105 different rooted topologies and a maximum probability of 0.95% to randomly get a particular root position by chance.
(2) The bootstrap support for the branch leading to that clade in the phylogenetic tree calculated from the whole data set is 100%: since the bootstrap value indicates the support the data have for a certain branch, we also require 100% support for the first direct descendants in the clade (Supplementary Appendix Fig. SA.1 available on Dryad at https://doi.org/10.5061/dryad.fj6q573rx). This requirement ensures that there is strong support in the data set for the root position of the clade when the entire data set is rooted with an outgroup.
(3) The site concordance factor (sCF) for the first direct descendants in the clade is significantly greater than 33%. The sCF is calculated by comparing the support of each site in the alignment for the different arrangements of the quartet around a certain branch. In other words, an sCF of 33% means equal support for any of the possible arrangements. Therefore, we require that the sCF of the deepest two levels of branches leading to that clade is significantly greater than 33%. Moreover, we require that the gene Concordance Factor (gCF) for the first direct descendants in the clade to be significantly greater than 33% of the sum of the gene concordance factor and the two Discordance Factors (gDF1 and gDF2). The gCF of a branch is calculated as the proportion of gene trees containing that branch, and gDFs are calculated as the proportion of gene trees containing one of the two other resolutions of that branch. Since for each branch in a bifurcating tree, there are three possible arrangements of clades around that branch, we ignore all gene trees that do not contain one of these arrangements (e.g., gene trees that contribute to neither the gCF nor the gDFs). Although there is no threshold regarding the required proportion of genes concordant with a certain branch, for convenience, we define branches with gCF significantly greater than 33% of the sum gCF$+$gDF1$+$gCF2 as branches that are concordant with enough genes in the alignment (Minh et al. 2020). To test whether the sCF and the gCF are significantly greater than 33%, we use a simple binomial test with a success probability of 0.33. The gCF, gDF1, gCF2, and sCF values are based on the tree estimated from the amino acid data set.
(4) At least 95% of the studies that have been published in the last decade support this clade: we searched google scholar for all published papers since 2009 that determine the root of the addressed clade. We then checked if at least 95% of those papers agree that the root position of the clade matches that in the ML tree we estimate from the whole data set (see Supplementary material available on Dryad).

### Estimating Unrooted Phylogenies

For the whole nucleotide and amino-acid data sets with ingroup and outgroup taxa, we inferred the unrooted phylogeny using IQ-TREE2 (Minh et al. 2020) with the best-fit fully partitioned model (Chernomor et al. 2016) and edge-linked substitution rates (Duchene et al. 2020). We then determined the best-fit reversible model for each partition using ModelFinder (Kalyaanamoorthy et al. 2017). See the algorithm for finding well-defined clades in Supplementary Appendix Algorithm SA.1 available on Dryad.

### Estimating Rooted Phylogenies

For each well-defined clade, we first removed all other taxa from the tree and then sought to infer the root of the well-defined clade using nonreversible models without outgroups. Using the best partitioning scheme from the reversible analysis, we inferred the rooted tree for each well-defined clade with the nonreversible models for amino acid (NR-AA) and nucleotide (NR-DNA) sequences (Minh et al. 2020). This approach fits a 12-parameter nonreversible model for DNA sequences, and a 380-parameter nonreversible model for amino acids. Details of the command lines used are provided in the Supplementary material section “Algorithm SA.2” available on Dryad. Each analysis returns a rooted tree. We performed 1000 nonparametric bootstraps of every analysis to measure the rootstrap support.

To assess the performance of the rootstrap and the ability of nonreversible models to estimate the root of the trees on smaller data sets, we also repeated every analysis on subsamples of the complete data set. For each well-defined clade, we performed analysis on the complete data set (100%) as well as data sets with 10%, 1%, and 0.1% of randomly selected loci from the original alignment.

### The Confidence Set of Root Branches using the Approximately Unbiased Test

In addition to the rootstrap support, we calculate the confidence set of all the branches that may contain the root of the ML tree using the approximately unbiased (AU) test (Shimodaira 2002). To do this, we reroot the ML tree with all possible placements of the root (one placement for each branch) and calculate the likelihood of each tree. Using the AU test, we then ask which root placements can be rejected in favor of the ML root, using an alpha value of 5%. We define the *root branches confidence set* as the set of root branches that are not rejected in favor of the ML root placement. An important difference between the AU test and the rootstrap support is that the AU test is conditioned on a single ML tree topology, but the rootstrap support is not. Because of this, they provide quite different information about the position of the root. The AU test assumes that the ML tree topology is true, and then seeks to determine the confidence set of root placements conditioned on that topology. The confidence set for the AU test will always therefore contain at least the ML root branch. The rootstrap does not assume any particular topology and instead asks how many times a particular root position appears across a set of bootstrap replicates. Because of this, it is possible for every branch in the ML topology to receive 0% rootstrap support. This can occur if none of the branches in the ML topology appear as the root branch in any of the bootstrap topologies.

### Reducing Systematic Bias by Removing Third Codon Positions and Loci that Fail the MaxSym Test

As it is common in many phylogenetic analyses to remove third codon positions from the alignment (Swofford et al. 1996), we wanted to assess the effect of removing third codon positions on the root inference and the rootstrap values in nucleotide data sets. For that purpose, we remove all the third codon positions from the nucleotide alignments and reran the analysis using the NR-DNA model.

Moreover, although the NR-AA and NR-DNA models relax the reversibility assumption, they still assume stationarity and homogeneity. To reduce the systematic bias produced by violating these assumptions, we used the MaxSym test (Naser-Khdour et al. 2019) to remove loci that violate those assumptions in the nucleotide and amino acid data sets and then reran all analyses as above.

### Applying the Methods to Two Clades Whose Root Position is Uncertain

In addition to the well-defined clades, we used the methods we propose here to infer the root of two clades of mammals whose root position is controversial; Chiroptera and the Cetartiodactyla.

There is a controversy around the root of the Chiroptera (bats) in literature. The two most popular hypotheses are: 1) the Microchiroptera-Megachiroptera hypothesis; where the root is placed between the Megachiroptera, which contains the family Pteropodidae, and the Microchiroptera, which contains all the remaining Chiroptera families. This hypothesis is well supported in the literature (Agnarsson et al. 2011; Meredith et al. 2011). However, more recent studies seem to provide less support for this hypothesis; 2) the Yinpterochiroptera-Yangochiroptera hypothesis, in which the Yangochiroptera clade includes most of Microchiroptera and the Yinpterochiroptera clade includes the rest of Microchiroptera and all of Megachiroptera. There is growing support for this hypothesis in the literature (Meganathan et al. 2012; Tsagkogeorga et al. 2013; Ren et al. 2018; Reyes-Amaya and Flores 2019).

Similar to Chiroptera, the root of Cetartiodactyla remains contentious in the literature. The three main hypotheses regarding the root of Cetartiodactyla are: 1) Tylopoda as the sister group for all other cetartiodactylans; 2) Suina as the sister group for all other cetartiodactylans; 3) the monophyletic clade containing Tylopoda and Suina as the sister group for all other cetartiodactylans.

To ascertain whether certain sites or loci had very strong effects on the placement of the root we follow the approach of Shen et al. (2017) and calculate the difference in site-wise log-likelihood scores ($\Delta $SLS) and gene-wise log-likelihood scores ($\Delta $GLS) between the supported root positions for each clade. Moreover, we analyzed subsamples of each data set to test the limits of using nonreversible models to root trees with smaller data sets.

## Results

### Inference of the Mammal Tree and Selection of Well-defined Clades

The trees inferred from the whole data sets with the nucleotide-reversible model and the amino-acid-reversible model (Supplementary Appendix Figs. SA.2, SA.3 and Table SA.2 available on Dryad) are consistent with the published tree (Liu et al. 2017). Five clades met all the criteria of well-defined clades, namely, Afrotheria, Bovidae, Carnivora, Myomorpha, and Primates in both amino acid and nucleotide data sets (see Supplementary Appendix Tables SA.1 and SA.2 available on Dryad). Trees in Newick format can be found on github: https://github.com/suhanaser/Rootstrap/tree/master/trees.

### High accuracy of the AA Nonreversible Model in Inferring the Root

**Figure 3.**
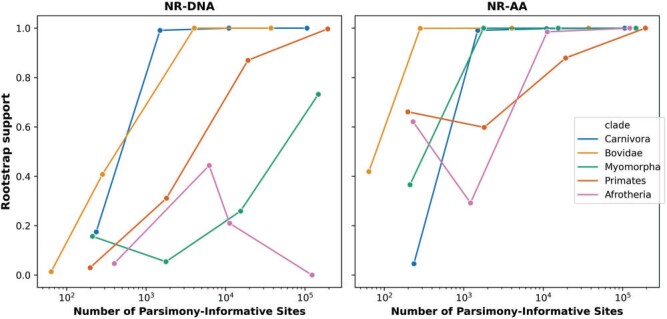
The rootstrap support value for each clade as a function of the number of parsimony-informative sites.

Using NR-AA, we inferred the correct root with very high rootstrap support for all five well-defined clades when all loci were used (Supplementary Appendix Table SA.3 available on Dryad). Moreover, for all the five clades, the true root was the only root placement in the confidence set of the AU test. The average running time of the NR-AA model (model estimation $+$ tree search $+$ bootstrap $+$ root inference) is 929 h on one core 2.6 GHz CPU. However, using the optimal number of cores for each data set reduced the average running time to 43.5 h per data set.

pt Our results show that using only 10% of the sites in the amino acid alignments (around 300,000 alignment columns) still gave very high rootstrap support values (>98%) for four of the five well-defined clades (Fig. 3) with no correlation between rSED and rBED and the size of the data set (Supplementary Table SA.3 available on Dryad). Moreover, in three of five well-defined clades, 1% of the sites (around 30,000 alignment columns) was enough to give a very high rootstrap support value for the assumed correct root placement. Using only 0.1% of the sites (around 3000 alignment columns) decreased the rootstrap support value noticeably in all data sets (Supplementary Appendix Table SA.3 available on Dryad). These values are shown for each data set in Figure 3, where the X-axis is plotted in terms of parsimony-informative sites to allow for a more direct comparison between data sets, and to assist those applying these methods in deciding whether to use them on their own data. Although the rootstrap support for the true root improves as the number of parsimony-informative sites increase, in some data sets (e.g., Afrotheria nucleotide data set) this is not the case (Fig. 3).

The nonreversible amino acid models were strongly preferred to the reversible models on the complete data sets (BIC values were 93,943 to 235,958 units better for the nonreversible models), and for the data sets with 10% of loci subsampled (BIC values were 3577 to 15,082 units better for the nonreversible models), but the opposite was true for the data sets 1% and 0.1% of the loci subsampled (e.g., BIC values were between 2102 and 2712 units worse for the nonreversible models for the 0.1% subsampled data sets; see Supplementary Table SA.7 available on Dryad for full results).

### Poor Performance of the DNA Nonreversible Model in Inferring the Root

We correctly inferred the root for four out of the five nucleotide data sets with the NR-DNA model, when all loci were used. However, the rootstrap support was generally lower than in the amino-acid data sets (Fig. 3, Supplementary Appendix Tables SA.3 and SA.4 available on Dryad). Similar to amino-acid data sets, there is no correlation between rSED and rBED and the size of the data set (Supplementary Table SA.4 available on Dryad). The average running time of the NR-DNA model (model estimation $+$ tree search $+$ bootstrap $+$ root inference) is 35.7 h on one core 2.6 GHz CPU and 4 h when the optimal number of cores for each data set were used.

In contrast to the NR-AA model, there is no conclusive preference for the NR-DNA model over the reversible DNA model for the data sets we analyzed (Supplementary Table SA.8 available on Dryad). In fact, the BIC values of the NR-DNA models are always worse than reversible models regardless of the size of the nucleotide data set except for three clades when all loci were included (Supplementary Table SA.8 available on Dryad). In two of the data sets (Myomorpha and Primates) where the NR-DNA model was better than the reversible model, the root placement was inferred correctly with high rootstrap support (>95%). In fact, the Afrotheria nucleotide data set is the only data set in which the nonreversible model was better than the reversible model but the root placement was inferred incorrectly.

Our results show that removing the third codon positions does not improve the rootstrap support value. In contrast, in some data sets removing third codon positions decreased the rootstrap support value and increased the rSED (Table 1).

**Table 1.** Rootstrap support and rSED values in whole nucleotide data sets and nucleotide data sets without third codon positions

	All loci		Without 3rd	
Clades	rootstrap (%)	rSED	rootstrap (%)	rSED
Afrotheria	0.0	2	0.0	2
Primates	99.7	0	90.1	0
Myomorpha	73.2	0	15.8	1
Carnivora	100.0	0	100.0	0
Bovidae	100.0	0	82.5	0

### Removing Loci that Violate the Stationarity and Homogeneity Assumptions Improves the rootstrap Support

As expected, our results show that removing loci that fail the MaxSym test improves the rootstrap support values when the rootstrap support value was less than 100% and/or the root placement was inferred incorrectly, as the case in some nucleotide data sets (Table 2).

**Table 2.** Rootstrap support values in whole data sets and data sets with loci that passed the MaxSym test only

	Amino Acid		Nucleotide	
Clade	All loci (%)	Passed MaxSym (%)	All loci (%)	Passed MaxSym (%)
Afrotheria	100.0	100.0	0.0	8.4
Primates	100.0	100.0	99.7	99.9
Myomorpha	100.0	100.0	73.2	88.3
Carnivora	100.0	100.0	100.0	100.0
Bovidae	100.0	100.0	100.0	100.0

### Microchiroptera–Megachiroptera or Yinpterochiroptera–Yangochiroptera?

**Figure 4.**
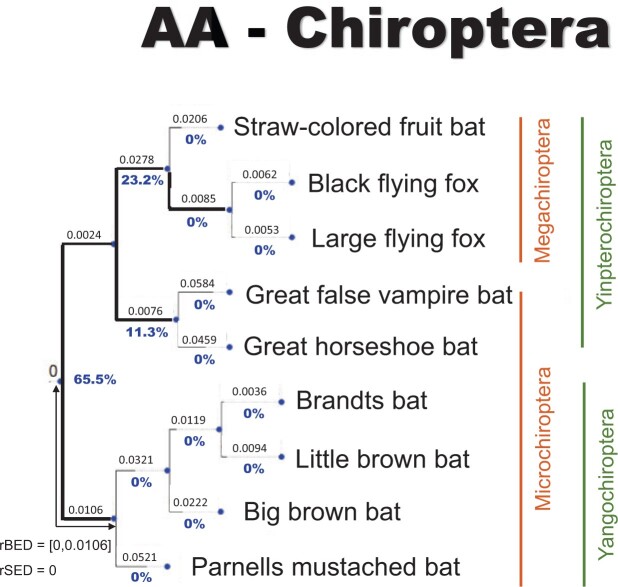
The ML rooted tree as inferred from the whole Chiroptera amino acid data set. Bold branches are branches in the AU confidence set. Blue values under each branch are the rootstrap support values.

Using the whole amino acid data set, our results show 65.5% rootstrap support for the Yinpterochiroptera–Yangochiroptera hypothesis and 23.2% for the Microchiroptera–Megachiroptera hypothesis. The remaining11.3% of the rootstrap support goes to supporting the branch leading to Rhinolophoidea as root branch of the bats (Fig. 4). Removing amino acid loci that fail the MaxSym test (110 loci) gives similar results, with 65.9% rootstrap support for the Yinptero-Yango hypothesis and 25.6% rootstrap support for the Micro-Mega hypothesis. In both cases, the AU test could not reject any of the three root positions that received nonzero rootstrap support (Supplementary Appendix Table SA.5 available on Dryad).

Using the NR-DNA model gives 100% rootstrap support for the Yinptero-Yango hypothesis, and we can confidently reject the Micro-Mega hypothesis in favor of the Yinptero-Yango hypothesis using the AU test (Supplementary Appendix Fig. SA.4 available on Dryad). Yet, removing nucleotide loci that fail the MaxSym test ($\sim$25% of the loci) decreases the support for the Yinptero-Yango hypothesis to 90.1%, although we can still confidently reject the Micro-Mega hypothesis using the AU test (Supplementary Appendix Table SA.5 available on Dryad).

**Figure 5.**
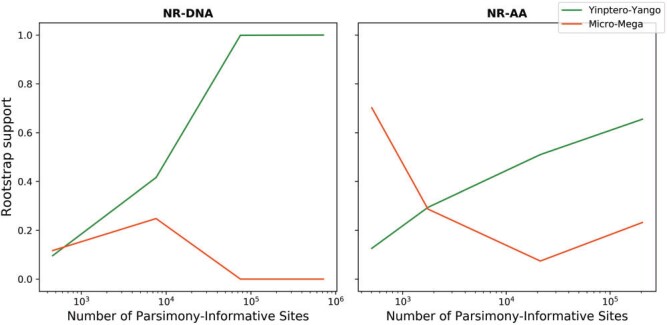
Rootstrap support value as a function of the number of parsimony-informative characters in the Chiroptera nucleotide and amino acid data sets using the nonreversible DNA model (NR-DNA) and the nonreversible amino acid model (NR-AA).

Interestingly, when we randomly subsample 10%, 1%, and 0.1% of the loci in the nucleotide data set, we consistently get the Yinptero-Yango hypothesis as the ML tree and the solely rooted topology in the AU confidence set (Supplementary Appendix Table SA.5 available on Dryad). Moreover, the rootstrap support value for the Yinptero-Yango hypothesis increases and the rootstrap support value for the Micro-Mega hypothesis decreases as more parsimony-informative sites are added to the alignment, for both nucleotide and amino acid data sets (Fig. 5, Supplementary Appendix Table SA.5 available on Dryad). These results are consistent with previous studies that used smaller data sets (Supplementary Appendix Fig. SA.10 available on Dryad).

The $\Delta $GLS and $\Delta $SLS values (Shen et al. 2017) reveal that approximately half of the nucleotide and amino acid loci prefer the Yinptero-Yango hypothesis while the other half prefers Micro-Mega hypothesis. Furthermore, slightly less than half of the nucleotide sites prefer the Yinptero-Yango hypothesis. However, more than two-thirds of the amino acid sites prefer the Yinptero-Yango hypothesis (Supplementary Appendix Fig. SA.5 available on Dryad). The distributions of $\Delta $GLS and $\Delta $SLS (Supplementary Appendix Fig. SA.6 available on Dryad) show that a small proportion of the amino acid loci ($\sim$1%) have very strong support for the Micro-Mega hypothesis, and removing those loci from the alignment increased the rootstrap support for the Yinptero-Yango hypothesis to 76.6%. Nonetheless, both root placements are still in the confidence set of the AU test (Supplementary Appendix Table SA.5 available on Dryad) with the amino acid data set. On the other hand, removing nucleotide loci with the highest absolute $\Delta $GLS value still gives the Yinptero-Yango hypothesis as the ML tree and the sole topology in the AU confidence set. Although the nucleotide data show a clear preference to the Yinptero-Yango hypothesis, in terms of BIC scores, the NR-DNA model performs worse than reversible models in all data sets except for the data set where we removed loci that failed the MaxSym test (Supplementary Table SA.5 available on Dryad). On the other hand, the NR-AA performs better than reversible models in big data sets (Supplementary Table SA.5 available on Dryad). Yet, the amino acid data do not allow us to distinguish between the two leading hypotheses for the placement of the root of the Chiroptera based on rooting with nonreversible models (Supplementary Table SA.5 available on Dryad).

### The Ambiguous Root of Cetartiodactyla

**Figure 6.**
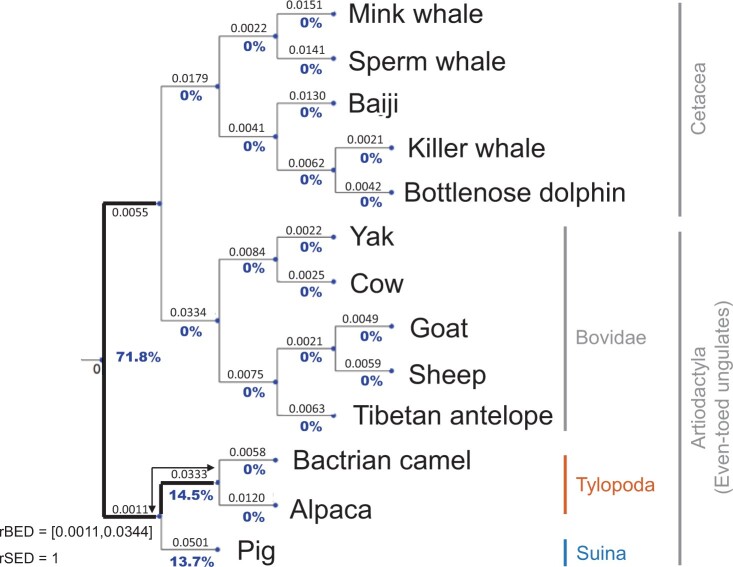
The ML rooted tree of as inferred from the whole Cetartiodactyla amino acid data set. Bold branches are branches in the AU confidence set. Blue values under each branch are the rootstrap support values.

The ML tree inferred with the whole amino acid data set places the clade containing Tylopoda (represented by its only extant family; Camelidae) and Suina as the sister group to all other cetartiodactylans with 71.8% rootstrap support (Fig. 6). Yet, The AU test did not reject Tylopoda alone as the sister group to all other cetartiodactylans. On the other hand, the ML tree inferred with the whole nucleotide data set places Tylopoda as the only sister group to all other cetartiodactylans with 71.0% rootstrap support, and we can confidently reject the Tylopoda $+$ Suina hypothesis using the AU test (Supplementary Appendix Fig. SA.7 available on Dryad).

Removing the amino acid loci that failed the MaxSym test ($\sim$1%) still places Tylopoda $+$ Suina as the sister group to all other cetartiodactylans, yet, it decreases the rootstrap support for the Tylopoda $+$ Suina hypothesis to 63.3% and increases the rootstrap support for the Tylopoda hypothesis to 28.5%. However, we still cannot reject either of the hypotheses using the AU test (Supplementary Appendix Table SA.6 available on Dryad).

Removing the nucleotide loci that failed the MaxSym test ($\sim$1%) still places Tylopoda as the only sister group to all other cetartiodactylans and the only rooted topology in the AU confidence set. However, it decreases the rootstrap support for the Tylopoda hypothesis to 68.7% and increases the rootstrap support for the Tylopoda $+$ Suina hypothesis to 20.1% (Supplementary Appendix Table SA.6 available on Dryad).

**Figure 7.**
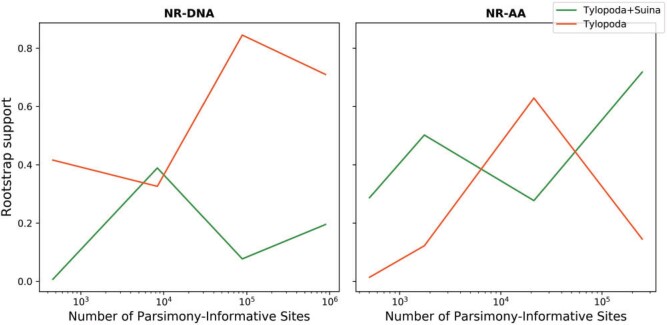
rootstrap support value as a function of the number of parsimony-informative 802 characters in the Cetartiodactyla nucleotide and amino acid datasets using the Non-Reversible 803 DNA model (NR-DNA) and the Non-Reversible Amino Acid model (NR-AA).

The results from the subsample data sets are mixed (Fig. 7). Analyses on smaller data sets show no clear pattern in the placement of the root (Supplementary Appendix Table SA.6 available on Dryad), leading us to conclude only that the analyses of the whole data set is likely to provide the most accurate result, but that it is plausible that adding more data may lead to different conclusions in the future.

$\Delta $ GLS analyses reveal that approximately, half of the amino acid and nucleotide loci favor the Tylopoda$+$Suina hypothesis, while the other half of loci favor the Tylopoda hypothesis (Supplementary Appendix Figs. SA.8 and SA.9 available on Dryad). On the other hand, two-thirds of the amino acid sites and more than 80% of the nucleotide sites favor the Tylopoda$+$Suina hypothesis. Removing 1% of the amino acid loci with the highest absolute $\Delta $GLS values still places Tylopoda $+$ Suina as the sister group to all other cetartiodactylans. However, the rootstrap support of the Tylopoda $+$ Suina decreased to 63.2% and the rootstrap support for the Tylopoda hypothesis remains approximately the same ($\sim$14.5%), while the rootstrap support for the Suina hypothesis increases from 13.7% to 22.4%. Yet, both the Tylopoda $+$ Suina hypothesis and the Tylopoda hypothesis are in the confidence set of the AU test, while the Suina hypothesis is rejected by the AU test (Supplementary Appendix Table SA.6 available on Dryad).

Removing 1% of the nucleotide loci with the highest absolute $\Delta $GLS values gives the Tylopoda$+$Suina as the sister group to all other cetartiodactylans with 39.7% rootstrap support. However, the sole rooted topology in the AU confidence set is the topology in which the root is placed on the branch leading to Suina (Supplementary Appendix Table SA.6 available on Dryad). Similar to Chiroptera and the well-defined clades, the NR-AA model performs better in terms of the BIC score than reversible models in big amino-acid data sets, while the NR-DNA performs worse than reversible models in all data sets (Supplementary Table SA.6 available on Dryad). We conclude that neither the nucleotide nor the amino acid data are adequate to confidently infer the root placement of Cetartiodactyla with nonreversible models.

## Discussion

In this article, we introduced a new measure of support for the placement of the root in a phylogenetic tree, the rootstrap support value, and applied it to empirical amino acid and nucleotide data sets inferred using nonreversible models implemented in IQ-TREE (Minh et al. 2020). The rootstrap is a useful measure because it can be used to assess the statistical support for the placement of the root in any rooted tree, regardless of the rooting method. In a ML setting, interpretation of the rootstrap support is similar to the interpretation of the classic nonparametric bootstrap. In a Bayesian setting, the same procedure could be used to calculate the posterior probability of the root placement given a posterior distribution of trees. It is noteworthy that the rootstrap support value is not a measure of the accuracy of the root placement and therefore should not be interpreted as such. However, it provides information about the robustness of the root inference with regard to resampling the data. This interpretation is consistent with the interpretation of the nonparametric bootstrap (Holmes 2003) but with regard to the root placement instead of the whole tree topology.

In addition to the rootstrap support value, we introduced another two metrics; the root branch-length error distance (rBED), and the  root split error distance rSED. Similar to the rootstrap metric, these additional metrics can be used in with any approach that generates rooted phylogenetic trees. We note that both metrics require the true position of the root to be known (or assumed) and that the rBED requires the rooting method to be able to accurately place the root in a specific position of the root branch.

In this study, we used these and other methods to assess the utility of nonreversible models to root phylogenetic trees in a ML framework. We focused on applying these methods to a large and very well curated phylogenomic data set of mammals, as the mammal phylogeny provides perhaps the best opportunity to find clades for which the root position is known with some confidence. As expected, our results show an exponential increase in the rootstrap support for the true root as we add more information to the MSA. Our results suggest that nonreversible amino-acid models are more useful for inferring root positions than nonreversible DNA models. One explanation for this difference between the NR-DNA and the NR-AA models is the bigger character-state space of the NR-AA models. These models have 400 parameters (380 rate parameters and 20 amino acid frequencies) whereas NR-DNA models have only 16 parameters (12 rate parameters and 4 nucleotide frequencies). This could allow the NR-AA model to capture the evolutionary process better than the NR-DNA model, potentially providing more information on the root position of the phylogeny. This hypothesis requires some further exploration though, and we note that the actual character-space of amino acids is much smaller than accommodated in NR-DNA models due to functional constraints on protein structure (Dayhoff et al. 1978).

Another explanation for the difference in performance between the NR-AA and NR-DNA models is that higher compositional heterogeneity in nucleotide data sets may bias tree inference. The fact that each amino acid can be specified by more than one codon, and that synonymous substitutions are more frequent than nonsynonymous substitutions, makes amino acid data sets less compositionally heterogeneous than nucleotide data sets. In principle, this bias can be alleviated by removing loci that violate the stationarity and homogeneity assumptions (Naser-Khdour et al. 2019). Our results suggest that this may be the case for the data sets we analyzed: we show that removing loci that violate the stationarity and homogeneity assumptions improves the accuracy and statistical support for the placement of the root. This is not surprising since the robustness of the rootstrap, similar to the bootstrap, relies on the consistency of the inference method, so removing systematic bias should improve its performance.

We used the nonreversible approach to rooting trees along with the rootstrap support to assess the evidence for different root placements in the Chiroptera and Cetartiodactyla. Using the amino acid data sets we found that in both cases, although there tended to be higher rootstrap support for one hypothesis, neither of the current hypotheses for either data set could be rejected. These results emphasize the importance of the rootstrap support value as a measure of the robustness of the root estimate given the data. In both the Chiroptera and Cetartiodactyla data sets the root placement varied among subsamples of the data set, and the rootstrap support reflects this uncertainty. However, checking the stability of root placement estimate by randomly subsampling from the whole Chiroptera data set show an obvious trend towards the Yinpterochiroptera–Yangochiroptera hypothesis as the data set increases in size. This trend is consistent with a small number of influential sites or loci having their signal progressively drowned out in favor of the Yinpterochiroptera–Yangochiroptera hypothesis as more data are added to the alignment. In both the Chiroptera and Cetartiodactyla cases, the amino acid data is inadequate to distinguish between certain root placements. On the other hand, in both the Chiroptera and Cetartiodactyla, the nucleotide data sets appear to show stronger support for a single root placement.

Comparing BIC scores of reversible and nonreversible models show that in most of the nucleotide data sets the reversible model was a much better fit to the data than the NR-DNA model. This is likely due to the limitations of the method we used to infer the NR-DNA model. Specifically, when inferring the trees with reversible DNA models, we used a partitioned model such that each partition was able to have an independent DNA substitution model. On the other hand, when we inferred the NR-DNA model we estimated a single model for the entire alignment. Thus, the NR-DNA model we inferred was unable to account for heterogeneity in the evolutionary process among partitions, possibly leading to its worse fit to the data when assessed using BIC scores. This suggests that using either mixture models or partitioned models may improve the fit of nonreversible DNA models to the data. The DNA results are consistent with results from previous study using the NR-DNA model and RootDigger (Bettisworth and Stamatakis 2020), although that study did not compare the performance of IQ-TREE and RootDigger on empirical data sets. Its results indicate that the NR-DNA model in IQ-TREE could not infer the correct root placement for any of the three tested data sets.

Our results demonstrate that the amino-acid nonreversible model can often be surprisingly accurate for inferring the root placement of phylogenies in the absence of additional information (such as outgroups) or assumptions (such as molecular clocks). In all of the well-defined clades that we examined, the nonreversible amino-acid model successfully identified the root that we identified a priori as correct, and with very high rootstrap support. Importantly, the nonreversible amino-acid models also tended to fit the data far better than their reversible counterparts did. Indeed, we show that root placements appear to be accurate even with data sets as small as 50 well-curated loci between fairly closely related taxa such as orders of mammals. Nevertheless, the application of the nonreversible amino acid models to two clades where the root position has previously been contentious failed to shed much additional light on the true root placement. Thus, while we show that the use of nonreversible models certainly has promise, we also show that it is no silver bullet.

Where a reliable outgroup taxon can be found, without the issues that can confound the inference of root placements using outgroups (Dalevi et al. 2001; Braun and Kimball 2002; Graham et al. 2002; Brady et al. 2006), we suggest relying on the use of outgroups. Nevertheless, where no reliable outgroups can be found, or where there is some reason to question the position of a root inferred using an outgroup (e.g., Bergsten 2005), our study suggests that using nonreversible models can provide a useful additional line of evidence for the position of the root of a phylogeny. We note also that the rootstrap value and the AU test could be used to provide estimates of the uncertainty of root placement using an outgroup taxon

Our work suggests a practical approach to inferring the root of a phylogenetic tree using nonreversible models. First, estimate an unrooted tree topology using the best reversible models available, excluding outgroup sequences. Next, fix the tree topology and use the best nonreversible models available to infer the ML root position of that tree. Finally, determine to what extent the ML root position should be trusted. The degree of trust that researchers should put in an inferred ML root position should be influenced by three factors (noting of course that all phylogenetic inferences are susceptible to be misled by model misspecification). First, the fit of the nonreversible model to the data should be better than the fit of the reversible model. This can be assessed using common criteria like AICc or BIC scores. A better fit of the nonreversible model provides some assurance that the data contain sufficient signal that using a nonreversible model is advisable in the first place. Our results show that the root placement was inferred correctly with high rootstrap support in 12 out of the 13 data sets in which the nonreversible model was preferable. In the absence of a better fit for a nonreversible model, we do not think any inferred ML root position should be trusted. Second, root positions with higher rootstrap support should be trusted more, because a higher rootstrap support indicates less variance among sites in the signal for the placement of the root. Third, ML root positions should be trusted more when the number of root placements included in the confidence set of an AU test is small, because a smaller confidence set indicates that there is less uncertainty in the root placement when the analysis is conditioned on the full alignment and the unrooted ML tree topology. A conservative approach to inferring root placements with nonreversible models would be to consider any root placement that has a substantial fraction of the rootstrap support and/or is included in the set of possible root placements identified by the AU test as a possible root placement given the assumptions of the model.

We hope that the combination of nonreversible models, rootstrap support, and AU tests will add another tool to the phylogeneticist’s arsenal when it comes to inferring rooted phylogenies.

Availability and implementation: rootstrap support is implemented in IQ-TREE 2 and a tutorial is available at the iqtree webpage http://www.iqtree.org/doc/Rootstrap. In addition, a python script is available at https://github.com/suhanaser/Rootstrap.

## Supplementary material

Data available from the Dryad Digital Repository: https://doi.org/10.5061/dryad.fj6q573rx.
